# Supplementary figures and images for: Hypertrophic cardiomyopathy mutations in the pliant and light chain-binding regions of the lever arm of human β-cardiac myosin have divergent effects on myosin function
Source: eLife. 2022 Jun 29;11:e76805. doi: 10.7554/eLife.76805 (PMC9242648; doi:10.7554/eLife.76805)

## Slide 1
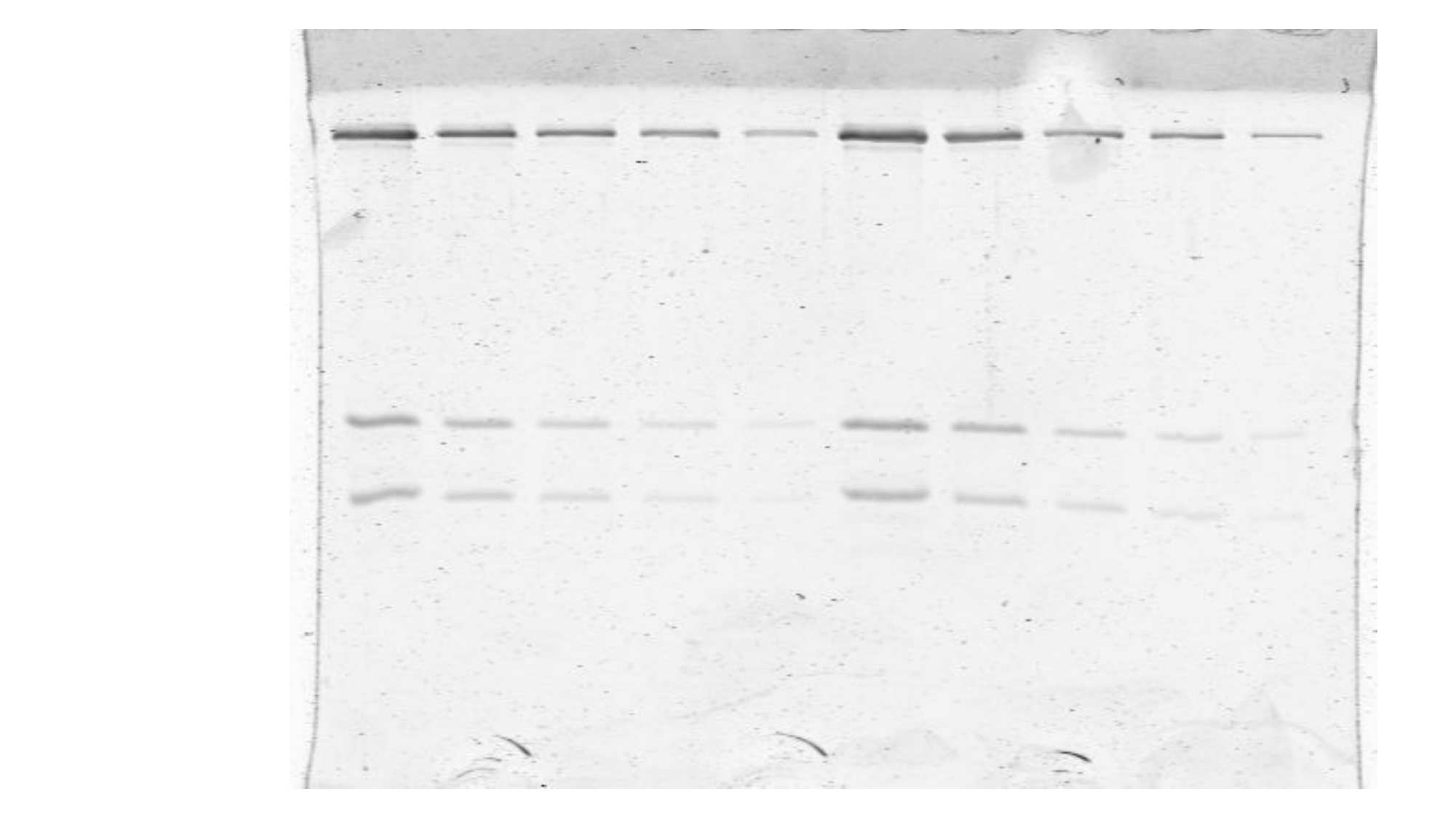

## Slide 2
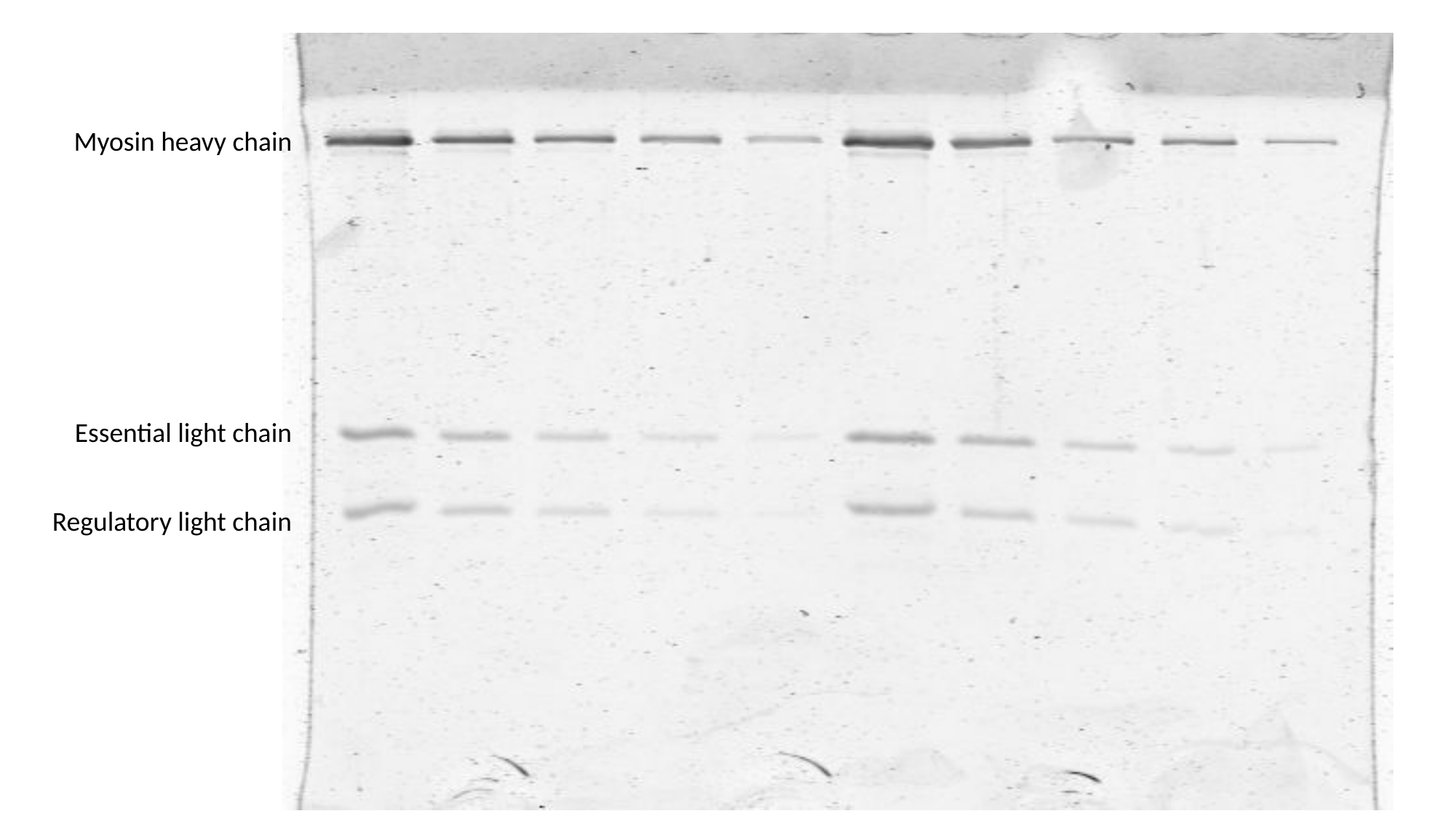

Myosin heavy chain
Essential light chain
Regulatory light chain

Supplement: Figure 2—source data 1. — Uncropped representative gel used to quantitate light chain loading (L781P 2-hep is shown) ± annotation. [file elife-76805-fig2-data1.pptx]
