# Supplementary material for: Hypertrophic cardiomyopathy mutations in the pliant and light chain-binding regions of the lever arm of human β-cardiac myosin have divergent effects on myosin function
Source: eLife. 2022 Jun 29;11:e76805. doi: 10.7554/eLife.76805 (PMC9242648; doi:10.7554/eLife.76805)
Supplement: Supplementary file 1. — For each mutation, two independent biological experiments were performed with freshly prepared myosins, where each biological replicate was measured with technical triplicates (6 total replicates for each protein). For WT 2- vs 25-hep, five biological replicates were measured with technical triplicates (15 total replicates for each protein). The 2-hep and 25-hep mutant myosins were prepared in tandem with a WT 2-hep control for comparison, given that actin-activated ATPase results show a slight drift from day-to-day. Results in the fourth and fifth columns, respectively, show the fitted values for kcat (s–1) and Kapp (μM) ± SE of the fit for each biological replicate. The kcat ratio of mutant 2-hep/WT 2-hep and mutant 25-hep/mutant 2-hep for each independent biological replicate is shown in the sixth and seventh columns, respectively, where the error is propagated from SE of the fit for each measurement. In the rightmost column (average mutant 25-hep/mutant 2-hep kcat ratio), statistically significant differences for mutant ratios vs WT ratio are shown, where * indicates p≤0.05, ** indicates p≤0.01, and *** indicates p≤0.001. [file elife-76805-supp1.pptx]

## Slide 1
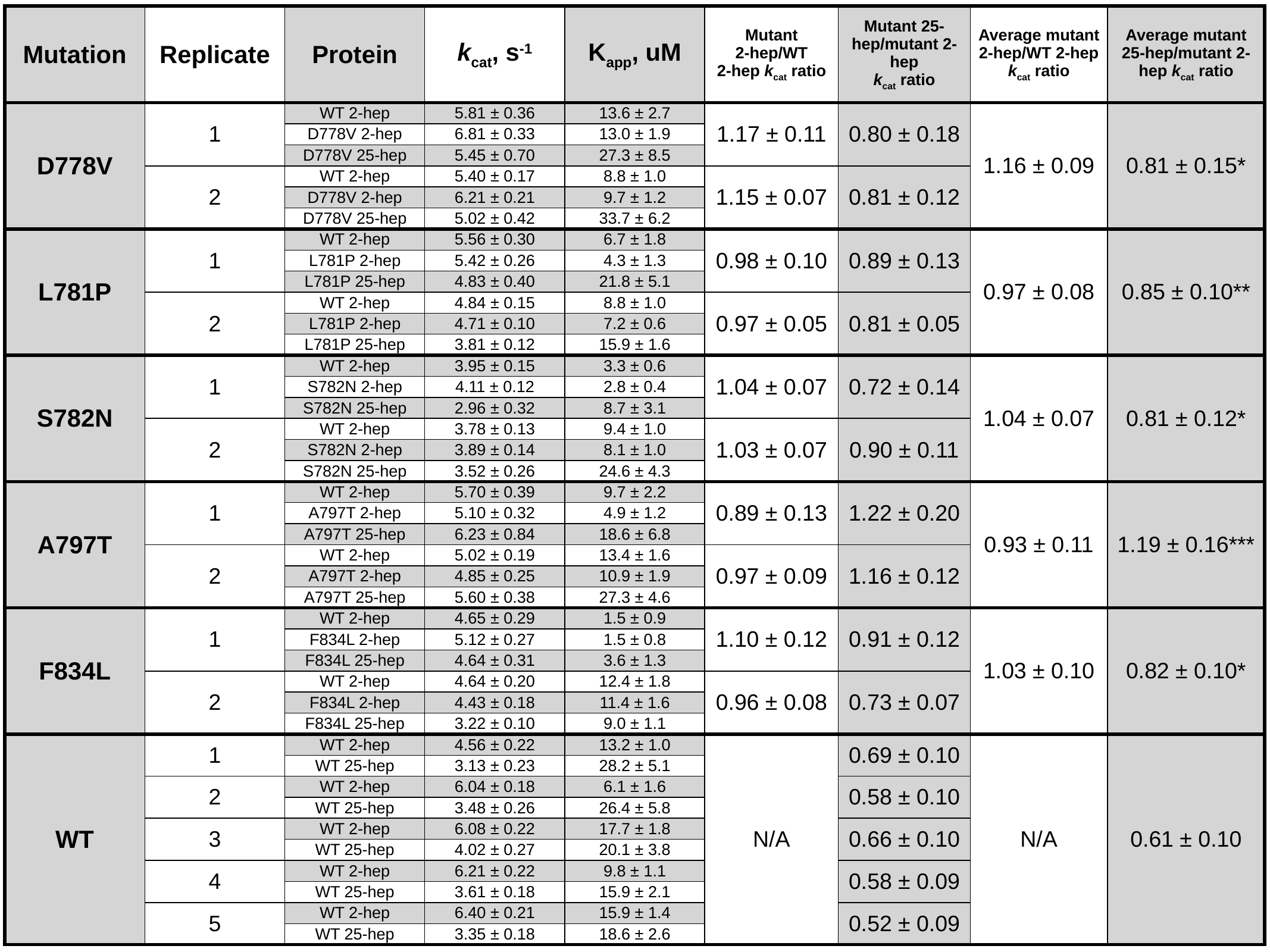

| Mutation | Replicate | Protein | kcat, s-1 | Kapp, uM | Mutant 2-hep/WT 2-hep kcat ratio | Mutant 25-hep/mutant 2-hep kcat ratio | Average mutant 2-hep/WT 2-hep kcat ratio | Average mutant 25-hep/mutant 2-hep kcat ratio |
| --- | --- | --- | --- | --- | --- | --- | --- | --- |
| D778V | 1 | WT 2-hep | 5.81 ± 0.36 | 13.6 ± 2.7 | 1.17 ± 0.11 | 0.80 ± 0.18 | 1.16 ± 0.09 | 0.81 ± 0.15\* |
| | | D778V 2-hep | 6.81 ± 0.33 | 13.0 ± 1.9 | | | | |
| | | D778V 25-hep | 5.45 ± 0.70 | 27.3 ± 8.5 | | | | |
| | 2 | WT 2-hep | 5.40 ± 0.17 | 8.8 ± 1.0 | 1.15 ± 0.07 | 0.81 ± 0.12 | | |
| | | D778V 2-hep | 6.21 ± 0.21 | 9.7 ± 1.2 | | | | |
| | | D778V 25-hep | 5.02 ± 0.42 | 33.7 ± 6.2 | | | | |
| L781P | 1 | WT 2-hep | 5.56 ± 0.30 | 6.7 ± 1.8 | 0.98 ± 0.10 | 0.89 ± 0.13 | 0.97 ± 0.08 | 0.85 ± 0.10\*\* |
| | | L781P 2-hep | 5.42 ± 0.26 | 4.3 ± 1.3 | | | | |
| | | L781P 25-hep | 4.83 ± 0.40 | 21.8 ± 5.1 | | | | |
| | 2 | WT 2-hep | 4.84 ± 0.15 | 8.8 ± 1.0 | 0.97 ± 0.05 | 0.81 ± 0.05 | | |
| | | L781P 2-hep | 4.71 ± 0.10 | 7.2 ± 0.6 | | | | |
| | | L781P 25-hep | 3.81 ± 0.12 | 15.9 ± 1.6 | | | | |
| S782N | 1 | WT 2-hep | 3.95 ± 0.15 | 3.3 ± 0.6 | 1.04 ± 0.07 | 0.72 ± 0.14 | 1.04 ± 0.07 | 0.81 ± 0.12\* |
| | | S782N 2-hep | 4.11 ± 0.12 | 2.8 ± 0.4 | | | | |
| | | S782N 25-hep | 2.96 ± 0.32 | 8.7 ± 3.1 | | | | |
| | 2 | WT 2-hep | 3.78 ± 0.13 | 9.4 ± 1.0 | 1.03 ± 0.07 | 0.90 ± 0.11 | | |
| | | S782N 2-hep | 3.89 ± 0.14 | 8.1 ± 1.0 | | | | |
| | | S782N 25-hep | 3.52 ± 0.26 | 24.6 ± 4.3 | | | | |
| A797T | 1 | WT 2-hep | 5.70 ± 0.39 | 9.7 ± 2.2 | 0.89 ± 0.13 | 1.22 ± 0.20 | 0.93 ± 0.11 | 1.19 ± 0.16\*\*\* |
| | | A797T 2-hep | 5.10 ± 0.32 | 4.9 ± 1.2 | | | | |
| | | A797T 25-hep | 6.23 ± 0.84 | 18.6 ± 6.8 | | | | |
| | 2 | WT 2-hep | 5.02 ± 0.19 | 13.4 ± 1.6 | 0.97 ± 0.09 | 1.16 ± 0.12 | | |
| | | A797T 2-hep | 4.85 ± 0.25 | 10.9 ± 1.9 | | | | |
| | | A797T 25-hep | 5.60 ± 0.38 | 27.3 ± 4.6 | | | | |
| F834L | 1 | WT 2-hep | 4.65 ± 0.29 | 1.5 ± 0.9 | 1.10 ± 0.12 | 0.91 ± 0.12 | 1.03 ± 0.10 | 0.82 ± 0.10\* |
| | | F834L 2-hep | 5.12 ± 0.27 | 1.5 ± 0.8 | | | | |
| | | F834L 25-hep | 4.64 ± 0.31 | 3.6 ± 1.3 | | | | |
| | 2 | WT 2-hep | 4.64 ± 0.20 | 12.4 ± 1.8 | 0.96 ± 0.08 | 0.73 ± 0.07 | | |
| | | F834L 2-hep | 4.43 ± 0.18 | 11.4 ± 1.6 | | | | |
| | | F834L 25-hep | 3.22 ± 0.10 | 9.0 ± 1.1 | | | | |
| WT | 1 | WT 2-hep | 4.56 ± 0.22 | 13.2 ± 1.0 | N/A | 0.69 ± 0.10 | N/A | 0.61 ± 0.10 |
| | | WT 25-hep | 3.13 ± 0.23 | 28.2 ± 5.1 | | | | |
| | 2 | WT 2-hep | 6.04 ± 0.18 | 6.1 ± 1.6 | | 0.58 ± 0.10 | | |
| | | WT 25-hep | 3.48 ± 0.26 | 26.4 ± 5.8 | | | | |
| | 3 | WT 2-hep | 6.08 ± 0.22 | 17.7 ± 1.8 | | 0.66 ± 0.10 | | |
| | | WT 25-hep | 4.02 ± 0.27 | 20.1 ± 3.8 | | | | |
| | 4 | WT 2-hep | 6.21 ± 0.22 | 9.8 ± 1.1 | | 0.58 ± 0.09 | | |
| | | WT 25-hep | 3.61 ± 0.18 | 15.9 ± 2.1 | | | | |
| | 5 | WT 2-hep | 6.40 ± 0.21 | 15.9 ± 1.4 | | 0.52 ± 0.09 | | |
| | | WT 25-hep | 3.35 ± 0.18 | 18.6 ± 2.6 | | | | |
