# Supplementary material for: Hypertrophic cardiomyopathy mutations in the pliant and light chain-binding regions of the lever arm of human β-cardiac myosin have divergent effects on myosin function
Source: eLife. 2022 Jun 29;11:e76805. doi: 10.7554/eLife.76805 (PMC9242648; doi:10.7554/eLife.76805)
Supplement: Supplementary file 2. — Single ATP turnover experiments were performed for both 2-hep and 25-hep myosins. Results show n, the number of independent experiments for each protein, average percent fast and slow phase ± SD, and average fast and slow rates ± SD. Stars denote statistically significant changes as compared to the WT control of the same protein construct, where * indicates p≤0.05 and **** indicates p≤0.0001. [file elife-76805-supp2.pptx]

## Slide 1
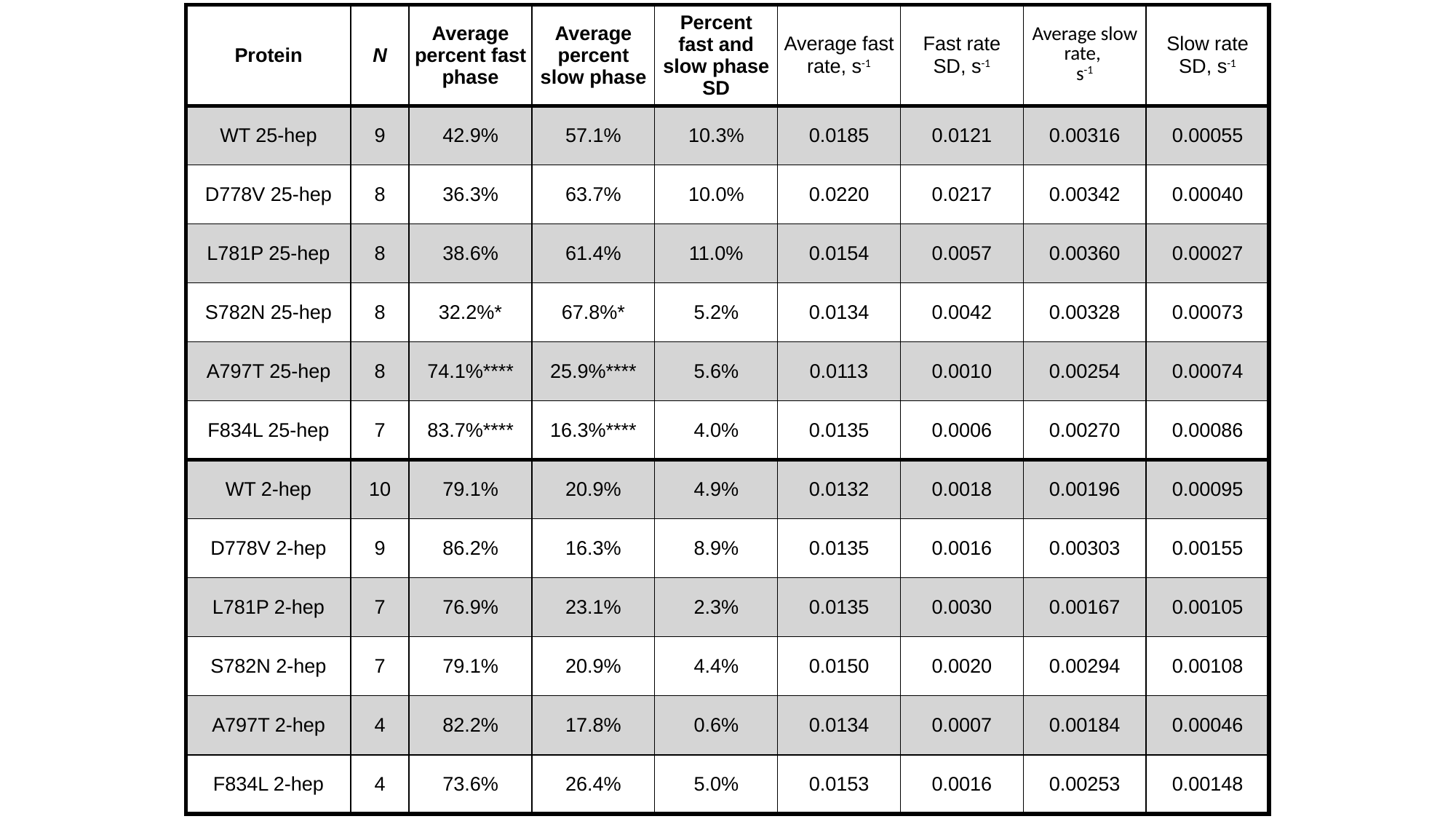

| Protein | N | Average percent fast phase | Average percent slow phase | Percent fast and slow phase SD | Average fast rate, s-1 | Fast rate SD, s-1 | Average slow rate, s-1 | Slow rate SD, s-1 |
| --- | --- | --- | --- | --- | --- | --- | --- | --- |
| WT 25-hep | 9 | 42.9% | 57.1% | 10.3% | 0.0185 | 0.0121 | 0.00316 | 0.00055 |
| D778V 25-hep | 8 | 36.3% | 63.7% | 10.0% | 0.0220 | 0.0217 | 0.00342 | 0.00040 |
| L781P 25-hep | 8 | 38.6% | 61.4% | 11.0% | 0.0154 | 0.0057 | 0.00360 | 0.00027 |
| S782N 25-hep | 8 | 32.2%\* | 67.8%\* | 5.2% | 0.0134 | 0.0042 | 0.00328 | 0.00073 |
| A797T 25-hep | 8 | 74.1%\*\*\*\* | 25.9%\*\*\*\* | 5.6% | 0.0113 | 0.0010 | 0.00254 | 0.00074 |
| F834L 25-hep | 7 | 83.7%\*\*\*\* | 16.3%\*\*\*\* | 4.0% | 0.0135 | 0.0006 | 0.00270 | 0.00086 |
| WT 2-hep | 10 | 79.1% | 20.9% | 4.9% | 0.0132 | 0.0018 | 0.00196 | 0.00095 |
| D778V 2-hep | 9 | 86.2% | 16.3% | 8.9% | 0.0135 | 0.0016 | 0.00303 | 0.00155 |
| L781P 2-hep | 7 | 76.9% | 23.1% | 2.3% | 0.0135 | 0.0030 | 0.00167 | 0.00105 |
| S782N 2-hep | 7 | 79.1% | 20.9% | 4.4% | 0.0150 | 0.0020 | 0.00294 | 0.00108 |
| A797T 2-hep | 4 | 82.2% | 17.8% | 0.6% | 0.0134 | 0.0007 | 0.00184 | 0.00046 |
| F834L 2-hep | 4 | 73.6% | 26.4% | 5.0% | 0.0153 | 0.0016 | 0.00253 | 0.00148 |
